# Relationships between full-day arm movement characteristics and developmental status in infants with typical development as they learn to reach: An observational study

**DOI:** 10.12688/gatesopenres.12813.2

**Published:** 2018-06-18

**Authors:** Joanne Shida-Tokeshi, Christianne J. Lane, Ivan A. Trujillo-Priego, Weiyang Deng, Douglas L. Vanderbilt, Gerald E. Loeb, Beth A. Smith

**Affiliations:** 1 Infant Neuromotor Control Laboratory, Division of Biokinesiology and Physical Therapy, University of Southern California, Los Angeles, California, 90089-9006, USA; 2 Department of Preventative Medicine, Division of Biostatistics, Keck School of Medicine, University of Southern California, Los Angeles, California, 90089-9234, USA; 3 Department of Pediatrics, Division of General Pediatrics, Keck School of Medicine, University of Southern California, Los Angeles, California, 90089-9234, USA; 4 Department of Biomedical Engineering, Viterbi School of Engineering, University of Southern California, Los Angeles, California, 90089, USA

**Keywords:** infant development, motor skills, wearable sensors, movement system, arm reaching

## Abstract

**Background:** Advances in wearable sensor technology now allow us to quantify the number, type and kinematic characteristics of bouts of infant arm movement made across a full day in the natural environment. Our aim here was to determine whether the amount and kinematic characteristics of arm movements made across the day in the natural environment were related to developmental status in infants with typical development as they learned to reach for objects using their arms.

**Methods:** We used wearable sensors to measure arm movement across days and months as infants developed arm reaching skills. In total, 22 infants with typical development participated, aged between 38 and 203 days. Of the participants, 2 infants were measured once and the other 20 infants were measured once per month for 3 to 6 visits. The Bayley Scales of Infant Development was used to measure developmental level.

**Results:** Our main findings were: 1) infant arm movement characteristics as measured by full-day wearable sensor data were related to Bayley motor, cognitive and language scores, indicating a relationship between daily movement characteristics and developmental status; 2) infants who moved more had larger increases in language and cognitive scores across visits; and 3) larger changes in movement characteristics across visits were related to higher motor scores.

**Conclusions:** This was a preliminary, exploratory, small study of the potential importance of infant arm movement characteristics as measured by full-day wearable sensor data. Our results support full-day arm movement activity as an area of interest for future study as a biomarker of neurodevelopmental status and as a target for early intervention.

## Introduction

Arm reaching skill develops at an early age ^1^. Reaching is a foundational, fundamental skill as it allows infants to touch and/or gain possession of and explore a desired object. In order to provide early intervention for infants who do not optimally develop these important foundational skills, it is crucial to quantify and describe infants’ earliest practice of spontaneous and goal-directed arm movements and their developmental progression of reaching skills. To accurately quantify practice, it is necessary to record arm movement behavior across full days. Our goal here was to determine how patterns and characteristics of spontaneous and goal-directed arm movements produced across full days relate to the development of reaching skill and overall developmental rate in infants with typical development. This is necessary background information that will begin to inform what type of early intervention (type and amount of practice) is required to improve developmental outcomes for infants at-risk of developmental delays.

Reaching skill changes rapidly in the first year. Across just a few months, the baby progresses from not reaching for objects to reaching and grasping an object using the whole hand, to progressing further to pick up a tiny pellet using a skilled grasp ^1^. Infants with typical development generally first learn to reach for objects at a very young age, usually between 3 and 5 months, with improvements made in straightness and smoothness during the first year ^2^,^4^. For example, in younger infants, Bhat and Galloway ^5^ reported on 13 infants, 8 weeks to onset of reaching, and described three phases of reaching. During the early phase, infants decreased their movement distance and velocity in the presence of a toy. During the mid-phase, infants increased the movement quantity, velocity, and smoothness; and decreased their hand–toy distance in the presence of a toy. During the late phase, infants continued to change their hand position to get closer and to contact the toy ^5^. Gonçalves *et al.* also studied young infants (aged 4–8 months) longitudinally and found an increased number of touches and hits, and changes in time and distance kinematics during reaching trials ^6^. Nelson *et al.* studied 11–14-month-olds (53 infants) and found improvements in reach straightness and smoothness, kinematic changes and emergence of handedness as the infants matured ^7^.

Although authors have described common general patterns occurring at each stage of reaching, infants proceed through the stages along their own unique timelines and developmental trajectories ^3^,^8^. While it is likely that the amount and type of arm movement practice an infant participates in across days and months contributes to their rate of reaching skill development, whether infants’ practice of arm movements across the day in their natural environment is related to the progression of reaching skill has not been investigated.

One reason this fundamental question about the relationship between arm movement practice and the development of reaching skill has not been investigated has been the lack of feasibility of collecting detailed full-day information about arm movements. As described in the aforementioned previous studies, previous assessment of reaching skill has been limited to short measurements in laboratory settings using three-dimensional motion analysis and video equipment. To allow full-day assessment, we have developed the use of wearable sensors to allow the measurement of full-day infant arm movement activity in the natural environment ^9^.

Advances in wearable sensor technology now allow us to quantify the number, type, and kinematic characteristics of bouts of infant arm movement made across a full day in the natural environment ^9^. In the current study, we used wearable sensors to quantify full-day arm movement characteristics across days and months as infants learned to reach. We used video coding of a standardized reaching assessment to describe reaching skill progression and the Bayley Scales of Infant Development ^1^ to measure developmental rate. Our aim here was to determine whether the amount and kinematic characteristics of arm movements made across the day in the natural environment was related to developmental status in infants with typical development as they learned to reach for objects using their arms.

## Methods

### Recruitment

We used wearable sensors to measure arm movement across days and months as infants developed arm reaching skills. In total, 22 infants with typical development participated, between 38 and 203 days of age ( Table 1). There were 2 infants measured once, with the other 20 infants measured once per month for 3 to 6 visits, until the infant successfully reached and grasped a toy with high skill (reaching skill assessment described below). Infants did not start the study at a particular time point in relation to their birthdate (e.g., near the day of the month they were born), however time between visits was kept consistent at 1 month +/- 7 days. This was a preliminary study to explore potential relationships of interest, and we used a sample of convenience. Inclusion criteria: infants were from singleton, full-term births (over 38 weeks). Exclusion criteria: infants experiencing complications during birth, or with any known visual, orthopedic or neurologic impairment at the time of assessment, or with a score at or below the 5 percentile for their age on the Alberta Infant Motor Scale ^10^ at the time of testing were excluded. Infants were recruited by a member of the research team in-person at the Eisner Health Clinic (Los Angeles, CA, USA), through fliers distributed or posted at the University of Southern California (USC), and by word of mouth. This study was approved by the Institutional Review Board of USC (HS-14-00690). A parent or legal guardian signed an informed consent form prior to their infants’ participation.

**Table 1.** Demographic characteristics of infants.

Gender	Ethnicity	Race	Highest education of either parent
10 male	16 Hispanic or Latino	10 White/Caucasian	5 doctorate
12 female	6 not Hispanic or Latino	9 other	5 high school
		1 American Indian/Alaska Native	3 bachelor
		1 Black or African American	3 some college
		1 declined	3 declined
			2 master
			1 associate

### Assessment

Infants were measured primarily in the family’s home. Per the family’s preference, two families came to the laboratory at the USC Health Science Campus for some of their visits. For these visits, they were in the laboratory for about an hour and then resumed their typical daily activities for the rest of the day while the baby wore the movement sensors. At each visit of the family to the laboratory or the researcher to the family’s home, the infant’s weight, body and limb lengths, and head and limb circumferences were measured. Motor, cognitive and language development were assessed by administering the Bayley Scales of Infant Development, 3 edition, a standardized, norm-referenced observational scale ^1^. In total, 5 min of video was recorded of the infant’s spontaneous movement in supine, while they wore a sensor on each arm. The parent or guardians’ highest level of education completed was recorded, as was the number of languages spoken in the home. Families were compensated for each visit. Data were stored on a password-protected server or in a REDCap electronic database (version 6.14.2) hosted by USC.

### Wearable sensors

**Figure 1.**
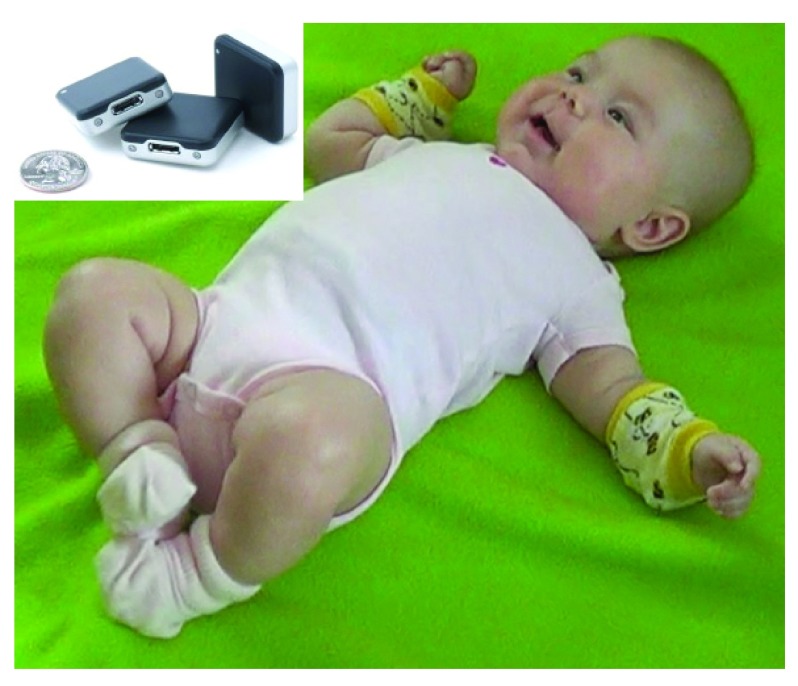
Infant wearing sensors on each arm. Inset: Three wearable sensors shown with a standard U.S. quarter dollar for reference.

Small, lightweight, wireless wearable sensors (APDM, Inc., Portland, OR, USA) were inserted into custom fabricated arm sleeves with a pocket to secure and cover the sensor and were placed above the infant’s wrists. The movement sensors are plastic and measure 48.5 × 36.5 × 13.5 mm and weigh 22 g ( Figure 1). The sensors were actively synchronized to each other, recording at 20 samples per second. The infant continued to wear the movement sensors for 8–13 h as the infant and caregiver(s) engaged in their normal daily activities. Caregivers were instructed to pursue their typical activities, and to remove the sensors for any water activities (e.g., bathing or swimming). The arm sleeves and sensors were removed by the caregiver when the infant went to bed for the night and were picked up by the research team the following day.

### Reaching skill assessment and electroencephalography

**Figure 2.**
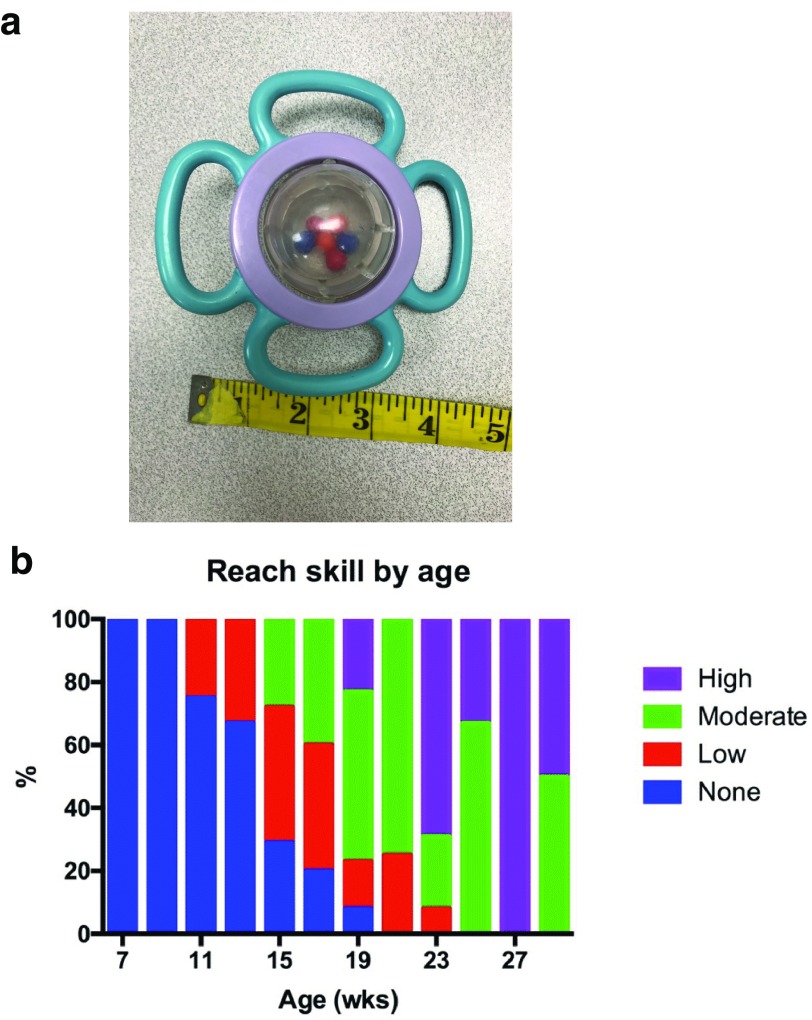
**a**. Object used in reaching skill assessment, shown with a measuring tape showing inches. Toy was rigid plastic, with rotating clear plastic center globe with balls that made noise when toy was moved. **b**. Percentage of visits that were assessed at each skill level, by chronological age.

At each session, we simultaneously assessed reaching skill and electrical activity of the brain using electroencephalography (EEG). We placed an EEG cap on the infant. One video camera was positioned to record object contacts during reaching. Infants sat the lap of their caregiver and were held securely around the trunk. There was a baseline EEG trial for 1 min, followed by 5 reaching trials alternating with 5 no-reach trials. Each reaching and no-reach trial lasted 20 s. For each reach trial, a small, graspable toy was positioned at mid-line, within the infant’s reach ( Figure 2a). If the infant successfully grasped the toy, they were allowed to explore it briefly before we removed it and offered it again, for the duration of the 20 s trial. The no-reach trials were 20 s of social interaction without an object in reach. For detailed EEG methods and results, please see our 2018 publication ^11^.

### Data analyses

*Wearable sensors*. We analyzed full-day arm movement data as described in our previous paper ^9^. We calculated the mean values across a full day for the following variables for right and left arm movement data. Descriptions of the measures are briefly summarized here. The daily arm movement rate *(*bouts/hours awake) is the number of bouts of arm movement an infant made across a full day, normalized to number of hours (to the nearest 5 min) that the infant was awake and wearing the movement sensors *.* A new bout of arm movement was counted each time the arm paused. A higher rate indicates that the infant moved more across the course of the whole day. We also calculated the duration (s), average acceleration (m/s ), and peak acceleration (m/s ) of each arm movement bout and reported the daily mean *.* Finally, as a general calculation of overall arm “activity”, we calculated the area under the absolute value of the resultant acceleration curve across the time period the sensors were worn by the infant. To compare between visits, we normalized to number of hours (to the nearest 5 min) that the infant was awake and wearing the movement sensors (normalized acceleration area). A larger normalized acceleration area value indicates that the infant is moving the arm more frequently and/or faster than a smaller value.

*Reaching skill assessment*. Video data of reaching behavior was behavior-coded by a single coder using ELAN frame-by-frame analysis software (version 4.6.2) ^12^. We identified when a reach was performed and the outcome of the reach attempt. Reaches were selected from the continuous video recording if the hand started from a still position or change in direction and moved closer to the toy being presented. The outcome of reaches was coded into four categories: no contact (infant was not close to contacting toy, for example got distracted and appeared to stop the attempt), miss (infant was clearly attempting to and close to touching toy but did not), touch (infant touched toy with fingers or palm of hand), or grasp (infant grasped toy with fingers or palm of hand) for each arm. Finally, we classified reaching skill at each visit as none, low, moderate, or high. None: the infant did not reach for the object, no touching or grasping. Low: the infant tried to reach for the object; however, there were only a few touches. Moderate: the infant reached for the object, but usually not right away, and the grasping was not mature. High: the infant reached directly and straight for the toy in almost all the trials, and the grasping was mature.

*Statistical analyses*. To determine whether the amount and kinematic characteristics of arm movements are related to developmental status, we calculated the intercept and slope (per day) for right and left arm movement variables (daily arm movement rate, duration, average acceleration, peak acceleration, normalized acceleration area) for each infant across days in age and then correlated these with Bayley composite scores (motor, cognitive or language). Bayley composite scores are determined in 2-week, age-normalized windows and created to have a range of 40–160, mean of 100 and SD of 15. Composite score classification are: 130 and above, very superior; 120–129, superior; 110–119, high average; 90–109, average; 80–89, low average; 70–79, borderline; 69 and below, extremely low ^1^. An infant developing at a steady rate would be expected to have composite scores that remained steady over time. Age was centered at the lowest age within the dataset (38 days), and the 2 infants with only one visit were not included in this analysis. Slope per day provided a summary of change over multiple visits. Statistical analyses were performed using IBM SPSS Statistics for Macintosh software (version 24.0).

## Results

### Reaching skill

Figure 2b shows the percentage of each reaching skill level demonstrated by infants at each chronological age, demonstrating that infants progress at different rates and there is not a direct relationship between chronological age and reaching skill. In general, we followed infants longitudinally across the time period when they progressed from no reaching skill to high reaching skill. In total, 11 infants started the study before they were able to reach, and subsequently moved from no skill to demonstrate some level of reaching skill by the second (6 infants), third (3 infants), or fourth (2 infants) visit. The other 11 infants started the study demonstrating some level of reaching skill. Reaching skill at each visit is provided in the supplementary data file.

### Relationships between full-day arm movement values and developmental status

**Figure 3.**
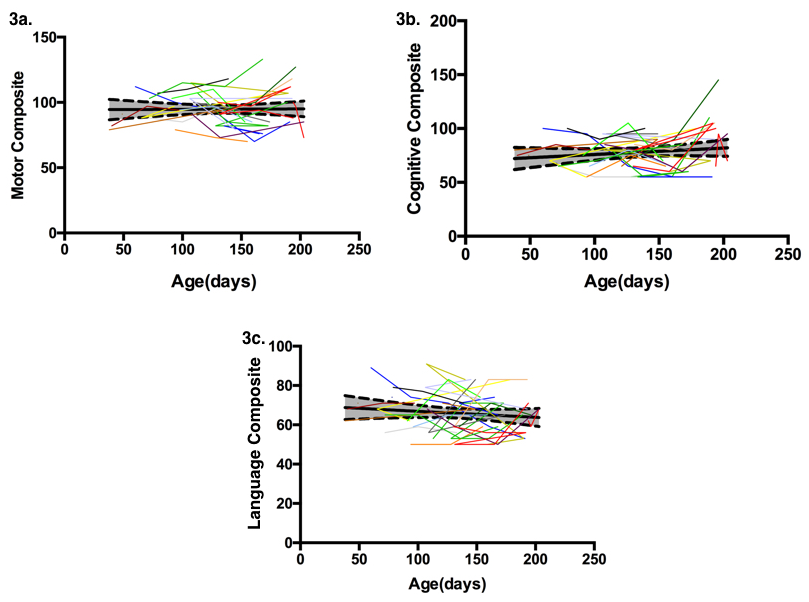
Bayley Scales of Infant Development scores by chronological age. Each colored line represents a different infant across repeated assessments. Single dots represent the two infants who were assessed only once each. The solid black line is the mean, the dashed black line is 1 standard deviation. ( **A**) Motor composite. ( **B**) Cognitive composite. ( **C**) Language composite.

Figure 3 shows Bayley composite scores (motor, cognitive, language) by age in days. Spearman correlations between arm movement variables (daily arm movement rate, duration, average acceleration, peak acceleration, normalized acceleration area) and Bayley composite scores (motor, cognitive or language) intercept (38 days of age) and slope (per day) are presented in Table 2. Correlations above 0.45 were identified as relationships of interest in this small sample and visualized with scatterplots. Right and left arms were evaluated separately, and differences between right and left arms reflect differences in the underlying variability of the respective data. Data from each visit are provided in the supplemental data file.

**Table 2.** Correlations between arm movement variables and Bayley composite score intercepts and slopes.

	Intercepts (at 38 days)			Slope (by day)		
	Motor	Language	Cognitive	Motor	Language	Cognitive
Intercepts (at 38 days)						
Bouts per awake time (left)	0.00	-0.32	-0.39	-0.01	0.37	0.39
Bouts per awake time (right)	-0.03	-0.55 *	-0.42	0.02	0.60 *	0.47 *
Mean duration (left)	-0.35	-0.31	0.03	0.29	0.23	0.08
Mean duration (right)	-0.11	-0.07	0.03	0.08	-0.02	0.08
Mean ave. acceleration (left)	-0.75 *	-0.43	-0.55 *	0.69 *	0.33	0.48 *
Mean ave. acceleration (right)	-0.45	-0.13	-0.34	0.39	0.02	0.24
Mean peak acceleration (left)	-0.64 *	-0.36	-0.36	0.54 *	0.21	0.32
Mean peak acceleration (right)	-0.32	-0.15	-0.32	0.23	-0.02	0.22
Area acceleration (left)	-0.48 *	-0.30	-0.12	0.52 *	0.33	0.26
Area acceleration (right)	-0.33	-0.30	-0.32	0.40	0.23	0.40
Slope (by day)						
Bouts per awake time (left)	-0.12	-0.06	0.19	0.04	0.00	-0.19
Bouts per awake time (right)	-0.04	0.23	0.35	-0.01	-0.28	-0.39
Mean duration (left)	0.32	0.32	-0.06	-0.26	-0.28	-0.03
Mean duration (right)	0.15	0.14	-0.06	-0.10	-0.08	-0.03
Mean ave. acceleration (left)	0.49 *	0.26	0.34	-0.5 *	-0.19	-0.33
Mean ave. acceleration (right)	0.22	0.00	0.14	-0.22	0.09	-0.04
Mean peak acceleration (left)	0.48 *	0.36	0.22	-0.45	-0.24	-0.23
Mean peak acceleration (right)	0.14	0.08	0.11	-0.07	0.04	0.01
Area acceleration (left)	0.29	0.04	-0.04	-0.42	-0.07	-0.09
Area acceleration (right)	0.15	0.07	0.13	-0.29	-0.02	-0.20

*Correlation above 0.45.

**Figure 4.**
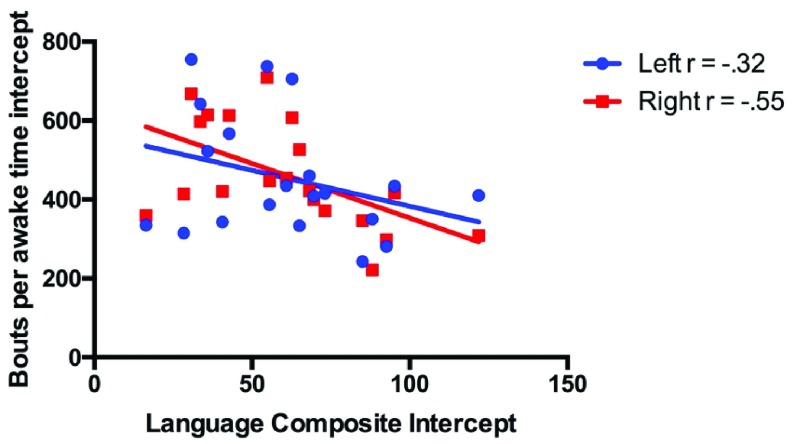
Scatter plot of bouts per awake time intercepts (for right and left arms) and Bayley composite language intercepts of each infant.

**Figure 5.**
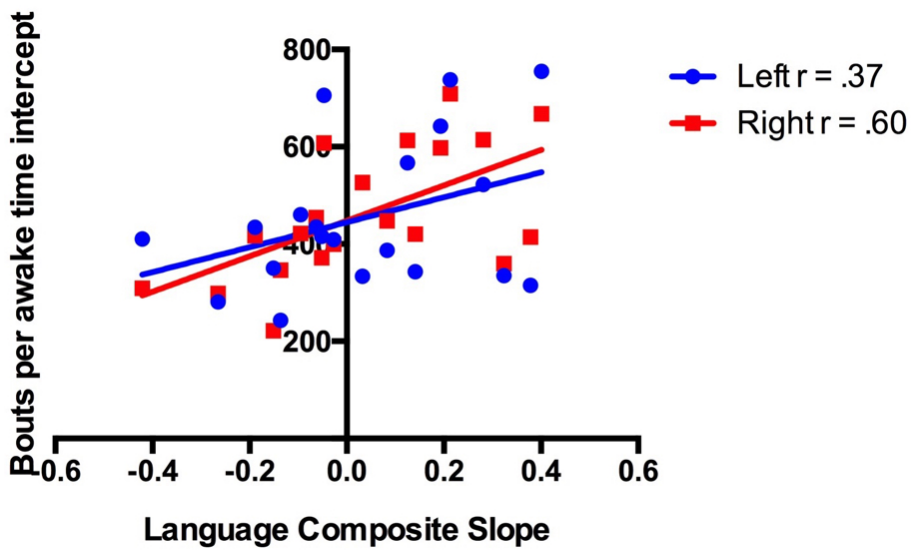
Scatter plot of bouts per awake time intercepts (for right and left arms) and Bayley composite language slopes of each infant.

**Figure 6.**
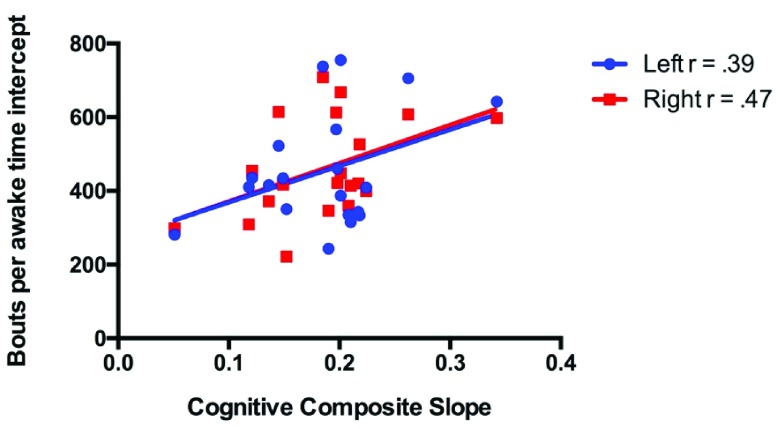
Scatter plot of bouts per awake time intercepts (for right and left arms) and Bayley composite cognitive slopes of each infant.

Bouts per awake time right arm intercept was negatively correlated with language intercept ( Figure 4), indicating that infants who moved their right arm more had lower language scores. The bouts per awake time right arm intercept was positively correlated with the language slope ( Figure 5) and cognitive slope ( Figure 6), indicating that infants who moved their right arm more had larger increases in language and cognitive scores across visits.

**Figure 7.**
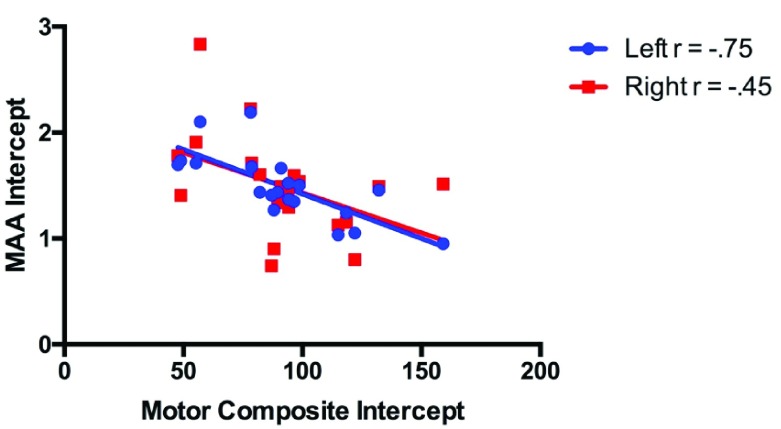
Scatter plot of mean average acceleration (MAA) intercepts (for right and left arms) and Bayley composite motor intercepts of each infant.

**Figure 8.**
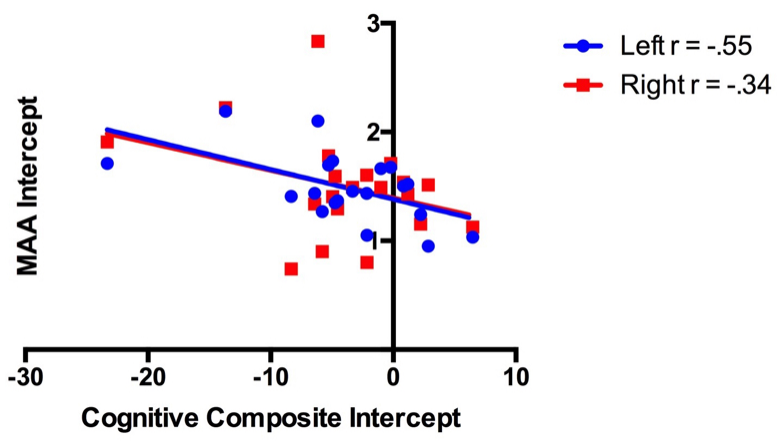
Scatter plot of mean average acceleration (MAA) intercepts (for right and left arms) and Bayley composite cognitive intercepts of each infant.

**Figure 9.**
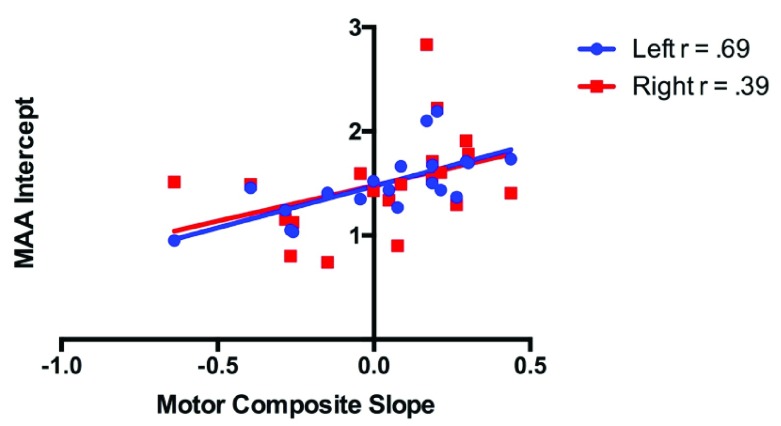
Scatter plot of mean average acceleration (MAA) intercepts (for right and left arms) and Bayley composite motor slopes of each infant.

**Figure 10.**
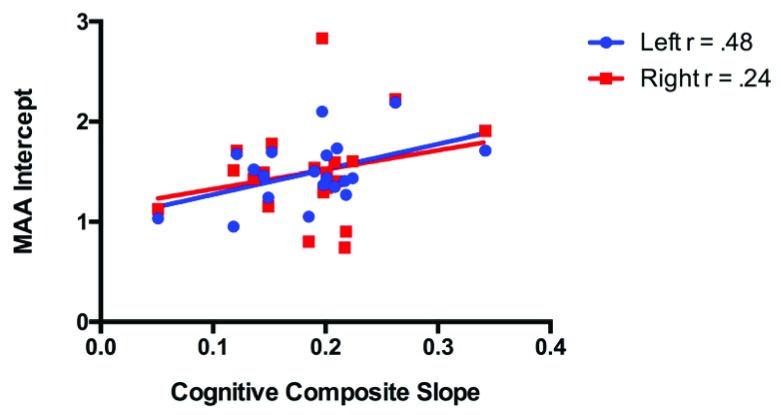
Scatter plot of mean average acceleration (MAA) intercepts (for right and left arms) and Bayley composite cognitive slopes of each infant.

The mean average acceleration left arm intercept was negatively correlated with the motor intercept ( Figure 7) and cognitive intercept ( Figure 8), indicating that infants who had higher average acceleration values of their left arm had lower motor and cognitive scores. The mean average acceleration left arm intercept was positively correlated with the motor slope ( Figure 9) and cognitive slope ( Figure 10), indicating that infants who had higher average acceleration values of their left arm had larger increases in motor and cognitive scores across visits.

**Figure 11.**
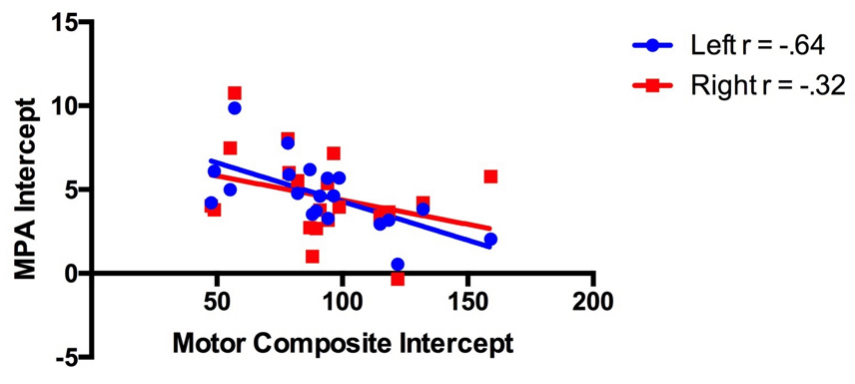
Scatter plot of mean peak acceleration (MPA) intercepts (for right and left arms) and Bayley composite motor intercepts of each infant.

**Figure 12.**
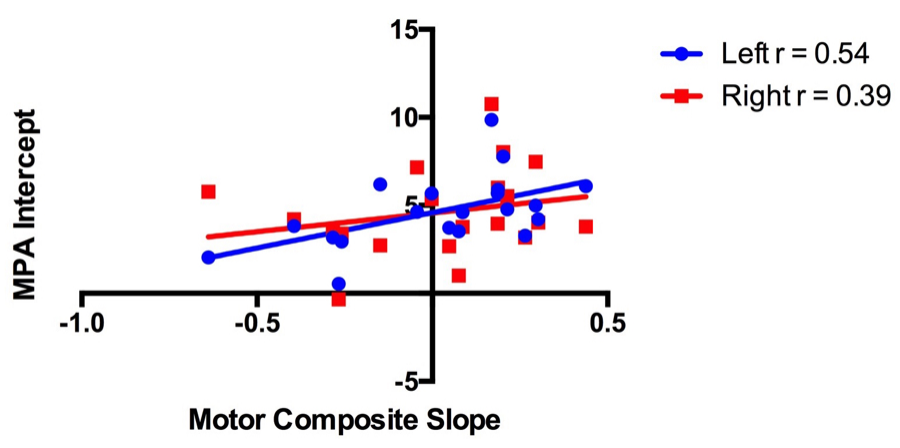
Scatter plot of mean peak acceleration (MPA) intercepts (for right and left arms) and Bayley composite motor slopes of each infant.

**Figure 13.**
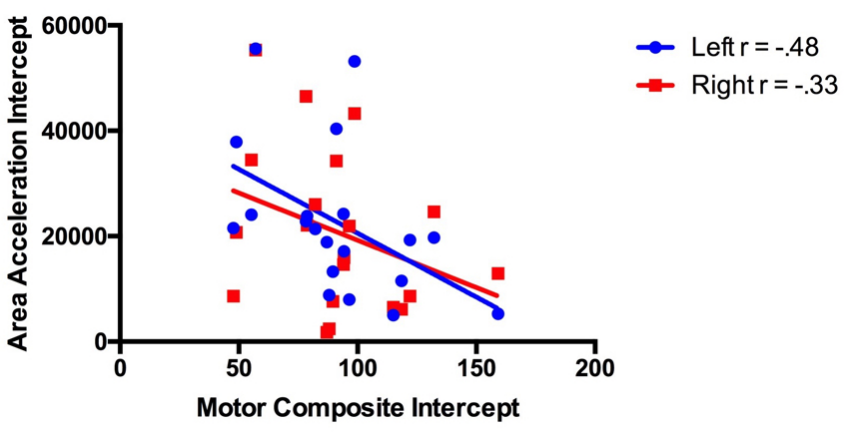
Scatter plot of normalized area of acceleration intercepts (for right and left arms) and Bayley composite motor intercepts of each infant.

**Figure 14.**
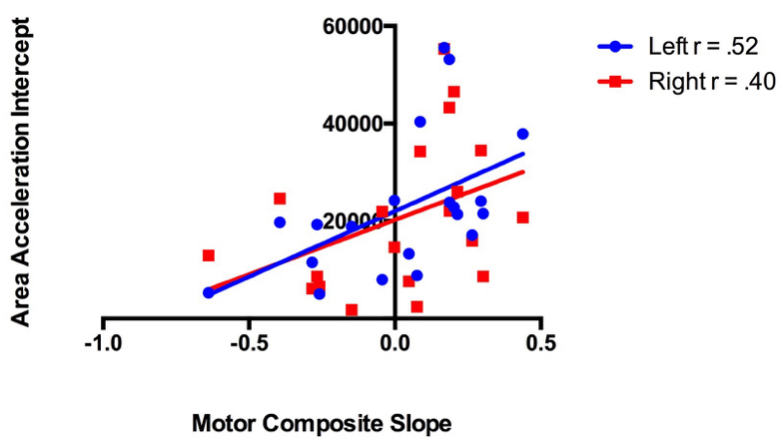
Scatter plot of normalized area of acceleration intercepts (for right and left arms) and Bayley composite motor slopes of each infant.

 The mean peak acceleration left arm intercept was negatively correlated with motor intercept ( Figure 11) and positively correlated with motor slope ( Figure 12), indicating that the infants who had lower peak acceleration values of their left arm had lower motor scores but larger increases across visits. The normalized acceleration area left arm intercept showed a similar pattern: a negative correlation with the motor intercept ( Figure 13) and a positive correlation with the motor slope ( Figure 14).

**Figure 15.**
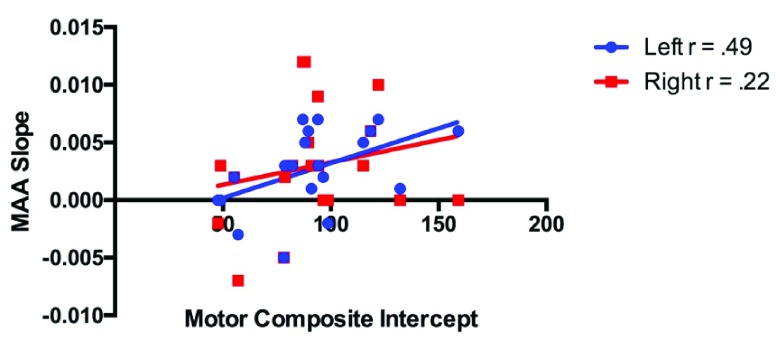
Scatter plot of mean average acceleration (MAA) slopes (for right and left arms) and Bayley composite motor intercepts of each infant.

**Figure 16.**
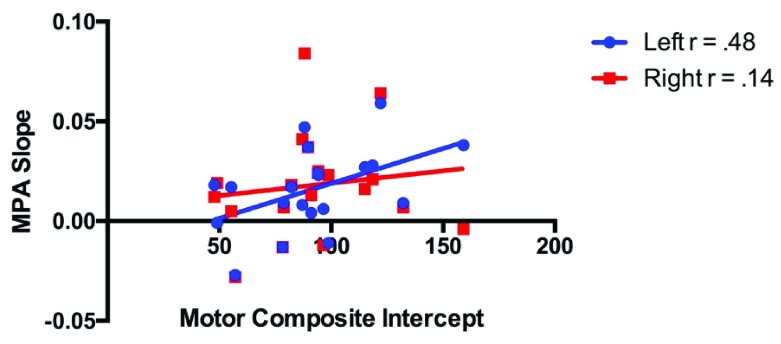
Scatter plot of mean peak acceleration (MPA) slopes (for right and left arms) and Bayley composite motor intercepts of each infant.

While the movement variable intercepts consider where an infant’s movement variables are in relation to the rest of the infants, the movement variable slopes consider how the infant’s movements change over time. Here, two correlations were significant: the motor intercept was positively correlated with mean average acceleration left arm slope ( Figure 15) and with mean peak acceleration left arm slope ( Figure 16). This indicates that infants who demonstrated larger increases in left arm average and peak acceleration values over time had higher motor scores.

## Discussion

Our main findings were: 1) infant arm movement characteristics as measured by full-day wearable sensor data were related to Bayley motor, cognitive, and language scores, indicating a relationship between daily movement characteristics and developmental status; 2) infants who moved more had larger increases in language and cognitive scores across visits; and 3) larger changes in movement characteristics across visits were related to higher motor scores.

### Movement as an input to the developing system

A relationship between detailed daily movement characteristics measured by wearable sensors and developmental status supports the potential of using wearable sensors to define the longitudinal trajectories of movement as an input to the developing nervous system. Wearable sensors provide a tool to measure the relationship between daily movement experience and the mastery of developmental milestones, giving us a window into experience-dependent plasticity of the developing brain and nervous system. Researchers have started to explore how the amount and type of practice in early infancy may support the scaffolding of development, for example early motor skills “unlocking” subsequent cognitive, motor and language skills, and vice versa ^13^,^17^. The idea is certainly not new, but the ability to quantify amount and type of motor experience is.

### Potential for early movement intervention

Our findings that infants who moved more had larger increases in language and cognitive scores across visits and that larger changes in movement characteristics across visits were related to higher motor scores support the role of early movement intervention to promote development. To be clear, we are not measuring cause and effect here, only associations. Previous work, though, has demonstrated that early motor training of reaching experience at 3 months affected the cognitive skills of object exploration and attention focusing 12 months later ^18^. Another study compared three groups of infants at 2.9 months of age, two intervention groups (14-day reaching intervention) and a control group (no intervention) ^19^. One intervention used a contingency design where the toy target moved and sounded upon contact only, thus incorporating both cognitive and motor aspects into the practice. The other intervention used a continuous design where the toy target moved and sounded continuously, independent of hand–toy contact, removing the cognitive contingent aspect. Results revealed that infants in the contingent group made the most progress in reaching skill over time compared to the two other groups ^19^. These findings highlight the importance of cognitive and motor interaction in early development and illustrate the potential for cascading effects on subsequent development initiated by early motor skills. Our next step is to start testing cause and effect by exploring the dose–response relationships between arm movement experience (amount and type of arm movement practice across days and weeks) and the development of reaching skill. It should also be noted, however, that higher cognitive scores may encourage greater exploration of the environment with the arms, resulting in larger changes in movement characteristics. This supports a potential role for intervening to promote cognitive development, with a potential positive impact on motor skill development. Determining what type of intervention will benefit a child at a given point in developmental time is a fundamentally important question that researchers are just starting to explore.

### Movement as an output of the developing nervous system

A relationship between detailed daily movement characteristics measured by wearable sensors and developmental status supports the potential of using wearable sensors to define the developmental trajectories of movement as an output of the developing nervous system. Movement patterns change as infants learn and grow. Infant developmental rates are highly variable, which makes it more challenging to identify atypical development early and accurately. Detailed records of infant behavior across long periods of time will provide insight into their capacity for movement in the natural environment, as opposed to their movement performance in a short period of time in a specific context. Wearable sensors provide a tool to quantify detailed characteristics of infant movement across days, and may support the development of an objective, quantified marker of atypical development. For example, wearable sensors provide a tool to measure the variability and repeatability of infant behavior over days and weeks, allowing us to test the theory that optimal variability (not too much and not too little) is a hallmark of a healthy neuromotor system ^20^,^24^.

### Bayley Scale scores

In general, infants showed variability in their rates of development over time. Individual infants deviated up and down from the composite score of 100 (see Figure 3), indicating that their rates of development sped up and slowed down, not remaining steady. If they remained steady, they would have been at or near a composite score of 100 at each measurement. Furthermore, we did not see regression to the mean. Visual inspection of Figure 3 does not show individual infant trajectories converging closer to a composite score of 100 over time. These findings fit with our knowledge that development is a complex, non-linear process ^25^,^26^, and support the theory that optimal variability (not too much and not too little) is a defining characteristic of a healthy neuromotor system ^20^,^24^.

Notably, our overall group composite cognitive and language scores, but not motor composite scores, appear lower than the anticipated mean, potentially due to a high number of infants in dual language and/or households with lower parent educational levels (see Table 1). Bilingual infant language development is acknowledged to have differences from monolingual development (for a review of this area, see 27). Early differences in infant development have not been explored, and whether older bilingual children have cognitive flexibility advantages or are equivalent to monolingual children is currently debated ^28^,^29^. Low socioeconomic status is also known to have a negative effect on infant developmental outcomes ^30^. The children in our study were scoring within the normal ranges on the Bayley scale (mostly low average to average; see Figure 3). However there could be an effect present already, as it is also known that living in poverty has negative effects in older, school-age children (for a review of this area, see 31).

### Limitations and future directions

This was a preliminary study in a small sample of infants. Our goal was to highlight potential relationships of interest to be pursued in future, larger, adequately powered studies. There are many potential factors that likely influence both movement characteristics and developmental rate. The amount of time in different positions (e.g., prone “tummy time”, potential restraint in car seats, being held and carried by caregivers), quality of caregiver–infant interaction, parenting style, cultural expectations, birth order, socioeconomic status, physical growth rate, nutritional status, amount and quality of sleep, personality/motivation, and even genetics are all potential contributing factors to examine. Understanding the relative contribution of each, as well as their responsiveness to intervention, will be key to providing early intervention to reach optimal developmental potential. This was a preliminary, exploratory, small study of the potential importance of infant arm movement characteristics, as measured by full-day wearable sensor data. Findings where only the right arm or left arm movement characteristics were correlated with developmental status variables likely reflect underlying variability in the data, and require further, adequately powered research to examine their importance. Our results support full-day arm movement activity as an area of interest for future study as a biomarker of neurodevelopmental status and as a target for intervention.

## Conclusions

Infant development is a complex process. We are starting to determine how and when we can intervene to have a positive impact on the important relationships between motor, cognitive and language development. Our findings here, of a relationship between detailed daily arm movement characteristics and developmental status, support the potential of using wearable sensors to trace out and classify the developmental trajectories of the nervous system.

## Data availability

A spreadsheet with full-day arm movement variables, reaching skill level, and Bayley scores for each participant at each assessment is available from figshare under a CC0 license at https://doi.org/10.6084/m9.figshare.6073886.v1 ^32^.

Data are available under the terms of the Creative Commons Zero “No rights reserved” data waiver (CC0 1.0 Public domain dedication).

## Consent

Written informed consent for the publication of the participants’ data and the identifying image of the infant wearing sensors ( Figure 1) was obtained from a parent or legal guardian of the participants.
