# Supplementary material for: Relationships between full-day arm movement characteristics and developmental status in infants with typical development as they learn to reach: An observational study
Source: Gates Open Res. 2018 Jun 18;2:17. Originally published 2018 Apr 5. [Version 2] doi: 10.12688/gatesopenres.12813.2 (PMC5915838; doi:10.12688/gatesopenres.12813.2)
Supplement: Peer review history

# Peer review: Referee response for version 1

**DOI:** 10.21956/gatesopenres.13880.r26355

**Published:** 2018-04-25

**Authors:** Regina T. Harbourne

**Affiliations:** 1 FAPTA Department of Physical Therapy, Rangos School of Health Sciences, Duquesne University, Pittsburgh, PA, USA

This is an interesting study, and foundational in terms of quantifying infant movement over time in typically developing children. This may serve as a basis for comparative evaluation of infants with delayed or atypical development in the future, and the wearable technology certainly has a great deal of potential.

 Methods:

 I am unclear on when the video assessment of reaching took place – could you please clarify that it took place in the home, and at the same time as the EEG testing, if that is true? Also, it would be helpful to know the reliability of the scoring for the reaching assessor (percent re-coded, percent agreement).

 Were the parents cautioned regarding activities during the day when the instrument was worn? I am curious about whether the parents treated the infants differently on days during instrumentation – more encouragement to move arms, less structured positioning (in infant seat or car seat). What instructions were provided?

 Results:

 I find it odd that a significant correlation would be found between only one arm (the left) for acceleration and motor Bayley. Does your video assessment of reaching provide any clues as to why this would be asymmetrical? We generally assume infants at this age will be symmetrical, so this stands out.

 Discussion:

 As an exploratory study, the relationships you found are interesting, and you make that point that correlation does not equal causation. However, your discussion does have a bias that movement drives cognition, rather than the opposite. I find that your statement here: “ infants who moved more had larger increases in language and cognitive scores across visits and that larger changes in movement characteristics across visits were related to higher motor scores support the role of early movement intervention to promote development” leads the reader to think in a particular direction. Giving equal time to the alternative possibility, that higher cognitive scores may encourage greater exploration of the environment with the arms, did not seem to come up as a part of the discussion. Perhaps you could add a little with this in mind.

 It is also possible that at these ages, the Bayley is not the most sensitive instrument to gauge cognitive skill. Other measures, such as look time or switch time within a habituation paradigm, may be more sensitive to long term predictors of cognition.

I have read this submission. I believe that I have an appropriate level of expertise to confirm that it is of an acceptable scientific standard.

# Response

**Published:** 2018-06-11

**Authors:** Beth A. Smith

Thank you for the insightful comments. We have revised the manuscript to provide more detail and add some discussion, as you suggested. This is an early exploratory study, and we hope that through our continued work and the work of others we are able to start answering some of the interesting questions you posed!

# Peer review: Referee response for version 1

**DOI:** 10.21956/gatesopenres.13880.r26353

**Published:** 2018-04-20

**Authors:** Ana Carolina de Campos

**Affiliations:** 1 Department of Physical Therapy, Child Development Analysis Laboratory (LADI), Federal University of São Carlos (UFSCar), São Carlos, Brazil

General comments: This is an interesting study using wearable sensors to describe whole day infant arm movements. The main strength is indicating the potential of measuring daily movements as a marker for developmental status. As an exploratory study, it opens possibilities for research seeking to understand infant development. I only have a few suggestions to improve clarity and relevance of the study.

 Methods:

 Table 1: Please revise table layout.  As it is, readers are led to follow the lines with the same color but information should not necessarily be read across lines.

 Regarding reaching assessments: were visits planned to happen at a certain age (e.g., near or at infant birth date every month)? This is relevant to increase reproducibility of the study. 

 Also, I wonder if authors have any information on compliance to the testing protocol, i.e., if infants actually wore the sensors during the planned hours. This would be helpful for other researchers planning full-day measurements of infant movements.

 Methods: Please provide more information on the object properties (e.g., rigidity, size in mm), as these have been describe to affect the quality of reaching.

 Discussion

 I missed a further exploration on the variability of reaching skill. It seems that only mentioning that development is not linear does not fully explain the increase in the frequency of “moderate ability” at the last assessment, since reaching is so well established by then.

 The results on the relation between arm variables and development are very interesting. I only recommend discussing in more detail possible reasons why the intercepts for bouts and acceleration indicate negative relations with language and motor/cognitive scores, respectively, as these results are counterintuitive.

 Concerning the Bayley scores, the authors mention a possible role of socioeconomic factors. Is there any information available on family income that could provide more support to these assumptions?

I have read this submission. I believe that I have an appropriate level of expertise to confirm that it is of an acceptable scientific standard.

# Response

**Published:** 2018-06-11

**Authors:** Beth A. Smith

Thank you for your thoughtful review and questions. We have revised the manuscript to provide more detail, as we were able. In regard to the Table formatting, that is handled by the journal, not us. We do not have official data on wearable sensor 'compliance', however visual inspection of the data shows consistent movement activity punctuated by periods of napping from the time we placed the sensors on until the caregiver reported removing them or the batteries ran out (whichever came first). It is possible that sensors were removed from the child and we interpreted this as napping, however caregivers overwhelmingly anecdotally reported that the sensors were not a nuisance and the infant wore them for the day without any problems. We hope to be able to test moderator/mediator variables in regard to developmental trajectories in future studies.

# Peer review: Referee response for version 1

**DOI:** 10.21956/gatesopenres.13880.r26370

**Published:** 2018-04-19

**Authors:** Christian Redd

**Affiliations:** 1 The Australian e-Health Research Centre, Commonwealth Scientific and Industrial Research Organisation (CSIRO), Herston, Qld, Australia

This research article describes an observational study to characterize the relationship between quantitative measures of full-day arm movements and developmental status in infants.

 Wearable sensors are an attractive avenue for developmental research, as they facilitate new avenues for ubiquitous and longitudinal measurements in the natural environment. Long-term wearable sensing has the potential to provide new metrics of developmental progression in the home to supplement assessments performed in the traditional clinical setting. Longer-term data in a more natural environment may be less susceptible to confounding influences inherent to short clinical examinations, such as behavioural state, stress, and unfamiliar sensory stimuli at the time of the examination.

 The authors recently published an algorithm to detect the quantity and kinematic characteristics of infant arm movements produced across a full day in their natural environments. This is an important first step for validating any new sensor technology, and a positive push for other infant sensing technologies. Many commercially available infant sensors purport to measure health characteristics in babies, but few have been validated towards their stated purpose and outcomes. The present study takes this prior work forward, by comparing outcomes from their algorithm against the validated Bayley Scales of Infant Development assessment tool.

 A few general comments follow that the authors may want to consider in the context of this study, or to incorporate into future infant wearable sensor efforts.

 The wearable sensor characteristics and their method of attachment is well described; however, some questions remain about their specific use. An assumption is made that they maintained their installed orientation throughout the day and were not moved or otherwise influenced. As the sensors were removed by the caregiver at the end of the day, a comparison to their initial orientation was not possible. A discussion of possible ways in which the sensors may be influenced over the course of the measurement period, and how those might be monitored and accounted for would provide greater confidence in the wearable sensor data.

 There is also a question of whether or not the presence of the wearable sensors influenced the movements of the infants while being worn. A prior research effort showed that weighting the arms and legs of 3-month old infants resulted in no change in either the quality or temporal organisation of fidgety spontaneous movements over a short study period ^1^. The sensors used in this study fall within the mass range of that prior result, but the measurement period is longer and the age range of infants studied is broader. Infants, especially at younger ages, may be susceptible to fatigue with the added weight of the study sensors and sleeves over long periods of wear time. It is difficult to determine what, if any, influence the presence of the sensors may have had on the movements recorded in this study.

   

 The algorithm used in the analysis is briefly summarised in this paper, but the internal rationale for a full-day worth of data collection is not well illustrated. This reviewer wonders what the sensitivity of the algorithm outputs are to total amount or time of data collected. Any time a sensor is on the infant, it presents a burden to them and their caregiver, and all attempts to minimise the overall assessment load should be taken. The data collected and presented within this study could potentially be expanded in such a sensitivity analysis to determine the relationship between the amount of data recorded and clinical assessment outcomes, towards an optimal measurement protocol.

 Following from the previous point, the wearable sensor outcomes report only averaged means, and individual normalized movement measures. While this is a reasonable approach for comparison between infants and visits, this seems to neglect the wealth of data provided by hours of sensing in the infant’s home environments. This reviewer would like to see more time-series analyses reported for the data collected; for instance, how movement characteristics change throughout the day, or even some classification of how daily movement bout distributions change longitudinally as infants age. While this may exceed the scope of the present publication, such analyses may better justify the use of wearable sensors over a full day measurement period.

 The authors acknowledge that this is a preliminary study in a sample of convenience, and that many factors potentially influence movement characteristics and developmental rate. The influence of external factors on the data recorded by the wearable sensors however, is not fully addressed. Manipulation and movement by caregivers, restraint in car seats or cribs, and other outside movements will all contribute to the acceleration and angular velocity data recorded by the wearable sensors. This signal noise is input along with meaningful movement bouts to the algorithm and may have some level of influence on the calculated outputs. In particular, the bout identification sensitivity of 90% ^2^ used a comparison to visual observation as the gold standard, over an interval of 20s of infant arm movement (n = 20 segments). This ground truth data is likely not fully representative of the hours of home environment data collection and potential sources (known and unknown) of outside noise. The authors acknowledge that the algorithm cannot differentiate between infant and caregiver-controlled movement, but do not measure or report on the ratio of intrinsic to extrinsic mandated movements. As babies mature, their interaction with their surroundings and caregiver evolve from spontaneous movements towards more goal-directed movements. It follows that the amount and distribution of external manipulation, restraint, and orientation controlled by the caregiver will also change. In a longitudinal study such as this one, the analytical methods (and their validation) must be robust to the evolution in these outside sources of movement noise during unsupervised data collection to ensure that reported outcomes are representative of  infant development.

 The study presented in this paper is a step towards long-term monitoring of infant movement characteristics. The main findings of the relationship between Bayley scores and full-day sensor data are intriguing and increases in language and cognitive scores corresponding to greater movements are a promising avenue for potential research directions and support efforts towards earlier interventions. Future efforts building on this small sample preliminary study, coupled with clarifications to the use and interpretation of wearable sensors, may better explain aspects of the complex process of infant development and provide new insights into individual trajectories.   

**Minor Comments:**

 In Figure 3, each subfigure has a different Y-axis range even though the individual Bayley Composites are assessed over the same range. Standardising the y-axis range would aid in interpreting the data.    

 During the reaching skills assessment, electroencephalography (EEG) was performed on the infant (Methods) but EEG data is not incorporated into any of analysis or reported in the results of this study. Another publication is cited as having detailed EEG methods and results, so it is not clear why it is included in the methods for this study.  

 In the analysis, the authors note that all reported sensor data was normalised to time that infant was awake. In the prior algorithm validation publication ^2^, it is reported that sleep time was visually estimated as periods of less than 3 movement bouts in 5 minutes. No citation or experimental rationale for this threshold is given, despite being used as a normalisation metric for all wearable sensor data. A more complete justification for the classification of sleep and awake periods would increase confidence in the metrics reported from the wearable sensors.

I have read this submission. I believe that I have an appropriate level of expertise to confirm that it is of an acceptable scientific standard.

## References

[1] DibiasiJEinspielerC: Load perturbation does not influence spontaneous movements in 3-month-old infants. *Early Hum Dev*.2004;77(1-2) : 10.1016/j.earlhumdev.2004.01.004 37-46 10.1016/j.earlhumdev.2004.01.004 15113630

[2] Trujillo-PriegoIALaneCJVanderbiltDLDengWLoebGEShidaJSmithBA: Development of a Wearable Sensor Algorithm to Detect the Quantity and Kinematic Characteristics of Infant Arm Movement Bouts Produced across a Full Day in the Natural Environment. *Technologies (Basel)*.2017;5(3) : 10.3390/technologies5030039 10.3390/technologies5030039 28824853PMC5558826

# Response

**Published:** 2018-06-11

**Authors:** Beth A. Smith

Thank you for the comprehensive and important comments. We agree with all of the points you raised. Some of them we are actively pursuing, others are areas we would like to pursue but do not currently have the resources to do so. We are open to collaboration with other researchers, and we could not agree more that these are important areas to pursue! One quick note: the orientation of the sensor likely does change throughout the day, at least slightly along the ventral/dorsal direction. While this would limit the ability to interpret the data from any one axis, our analysis uses the resultant of the 3 axes, so migration in orientation of the sensor in this way will not affect our results here. If the sensor moves more proximal or distal along the wrist, acceleration values may be affected. Per observation, the ventral/dorsal migration does occur but the proximal/distal generally does not when the sensors are attached using our method here. This has not, however, been systematically evaluated.
